# GaborNet: investigating the importance of color space, scale and orientation for image classification

**DOI:** 10.7717/peerj-cs.890

**Published:** 2022-02-25

**Authors:** Richard M. Rimiru, Judy Gateri, Micheal W. Kimwele

**Affiliations:** Jomo Kenyatta University of Agriculture and Technology, Nairobi, Kenya

**Keywords:** CBIR, Color space, Gabor filter, Convolutional neural networks, GaborNet

## Abstract

Content-Based Image Retrieval (CBIR) is the cornerstone of today’s image retrieval systems. The most distinctive retrieval approach used, involves the submission of an image-based query whereby the system is used in the extraction of visual characteristics like the shape, color, and texture from the images. Examination of the characteristics is done for ensuring the searching and retrieval of proportional images from the image database. Majority of the datasets utilized for retrieval lean towards to comprise colored images. The colored images are regarded as in RGB (Red, Green, Blue) form. Most colored images use the RGB image for classifying the images. The research presents the transformation of RGB to other color spaces, extraction of features using different color spaces techniques, Gabor filter and use Convolutional Neural Networks for retrieval to find the most efficient combination. The model is also known as Gabor Convolution Network. Even though the notion of the Gabor filter being induced in CNN has been suggested earlier, this work introduces an entirely different and very simple Gabor-based CNN which produces high recognition efficiency. In this paper, Gabor Convolutional Networks (GCNs or GaborNet), with different color spaces are used to examine which combination is efficient to retrieve natural images. An extensive experiment using Cifar 10 dataset was made and comparison of simple CNN, ResNet 50 and GCN model was also made. The models were evaluated through a several statistical analysis based on accuracy, precision, recall, F-Score, area under the curve (AUC), and receiving operating characteristic (ROC) curve. The results shows GaborNet model effectively retrieve images with 99.68% of AUC and 99.09% of Recall. The results also shows different images are effectively retrieved using different color space. Therefore research concluded it is very significance to transform images to different color space and use GaborNet for effective retrieval.

## Introduction

Content-Based Image Retrieval (CBIR) is considered a significant application in the area of computer vision.

**Figure 1:**
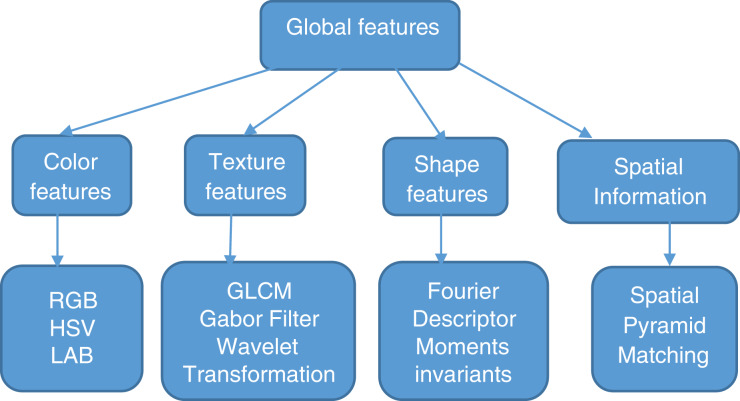
Classification of global features.

Many techniques have been invented to transform high-level concepts in images to features. These features are the basis for CBIR. Features are categorized into global and local features depending on the feature extraction techniques. Figure 1 shows classification of global features. Global features are color, texture, shape, and spatial information which gives a representation for the entire image. Global features are robust in feature extraction and similarity computations (Datta et al., 2008).

The process of retrieval of the appropriate images is usually followed by the extraction of discriminating attributes that best outlines the database images. Thus, retrieving the images depends on the comparing the already captured images that represents the significant features of the images rather than comparing it to the entire images. Researchers have developed many techniques to use these features for accurately retrieving the needed images from the databases since this is a crucial requirement for the domains. The majority of the datasets utilized for retrieval lean towards to comprise of colored images. The colored images are regarded as in RGB form. Most colored images use the RGB image for classifying the images since the latest models developed fails to achieve a color space change to the image (Zeiler & Fergus, 2014).

The research introduce the color space combined with texture for extraction features. Texture in the domain of processing images that provides details about the neighborhood spatial grouping of colors or the color intensity in an image. Images that have similar texture properties should therefore have the same spatial arrangements of colors or intensities, but not necessarily the same colors. Gabor filter is one of the techniques that extract image features using texture classification. Gabor eliminates the vast majority of the dissimilarity in lightning and contrast and reduces interpersonal dissimilarity.

The research propose conversion of different color space to investigate the efficiency in retrieval using natural images from Cifar 10 dataset. The research agrees with Chandana et al. (2017) in combining more than one feature for better performance and therefore proposed to use color and texture to extract image feature. The research extract features using RGB, transform RGB to different color spaces and use those features from different color space as an input to extract texture features.

### Contribution

In this paper, we propose using various color spaces and Gabor filter to extract image features and CNN to match similarity. The CNN will use Gabor filters as an input layer and keep on adjusting the parameters of Gabor filter to achieve the effective parameters.

The contributions of this paper are summarized as follows:

- The research is able to achieve the optimal Gabor parameters.
- The research obtain the state-of-art performance on various Gabor filter parameters.
- The research is able to achieve the performance of different color space through simple CNN, ResNet 50 and GaborCNN. It was observed different color space retrieve effectively different types of images in Cifar 10 and therefore it’s recommended to transform colors when extracting images to obtain effective retrieval.

### Color spaces

Color is seen as a sensation and not a characteristic measurement like length or temperature, however, the electromagnetic beam of noticeable frequency is quantifiable as a characteristic amount, thus there is a proper type of portraying for numerical prerequisites.

In human perspective of color, these various requirements can’t accommodate both, that is why the characterization used differ in accordance to the processing target used in the application. Color spaces alludes to fit frameworks whereby values are addressed according to the conventional color scheme as per the International Lighting Com-mission (CIE) standard. The color spaces are separated red, green and blue colors (RGB) data, the three essentials colors can’t be managed in numerical and specialized processors. It doesn’t give an exact portrayal of the remainder of the subsequent colors so fragmentation of the images is utilized to the initial state without colors, that is, highly contrasting (Luminance Y) and it is treated as light power dependent on the grayscale among high contrast as on account of TV broadcasting is modulated independently (Luan et al., 2018).

The procedure of processing colors, precision and the clarity relies upon the idea of the fundamental elements of the image and techniques for representing numerically and technically in the transmitter and recipient making it indistinguishable from the initial regular state motivated by them, and that the default state is the lighting component and two-shading qualities and to add other complementing mixtures which can prompt a critical improvement in execution and is balanced by the expansion in the involvement of numerical representation which yields a technical output (on account of adaptive color space) (Harman, 2002).

#### RGB color space

**Figure 2:**
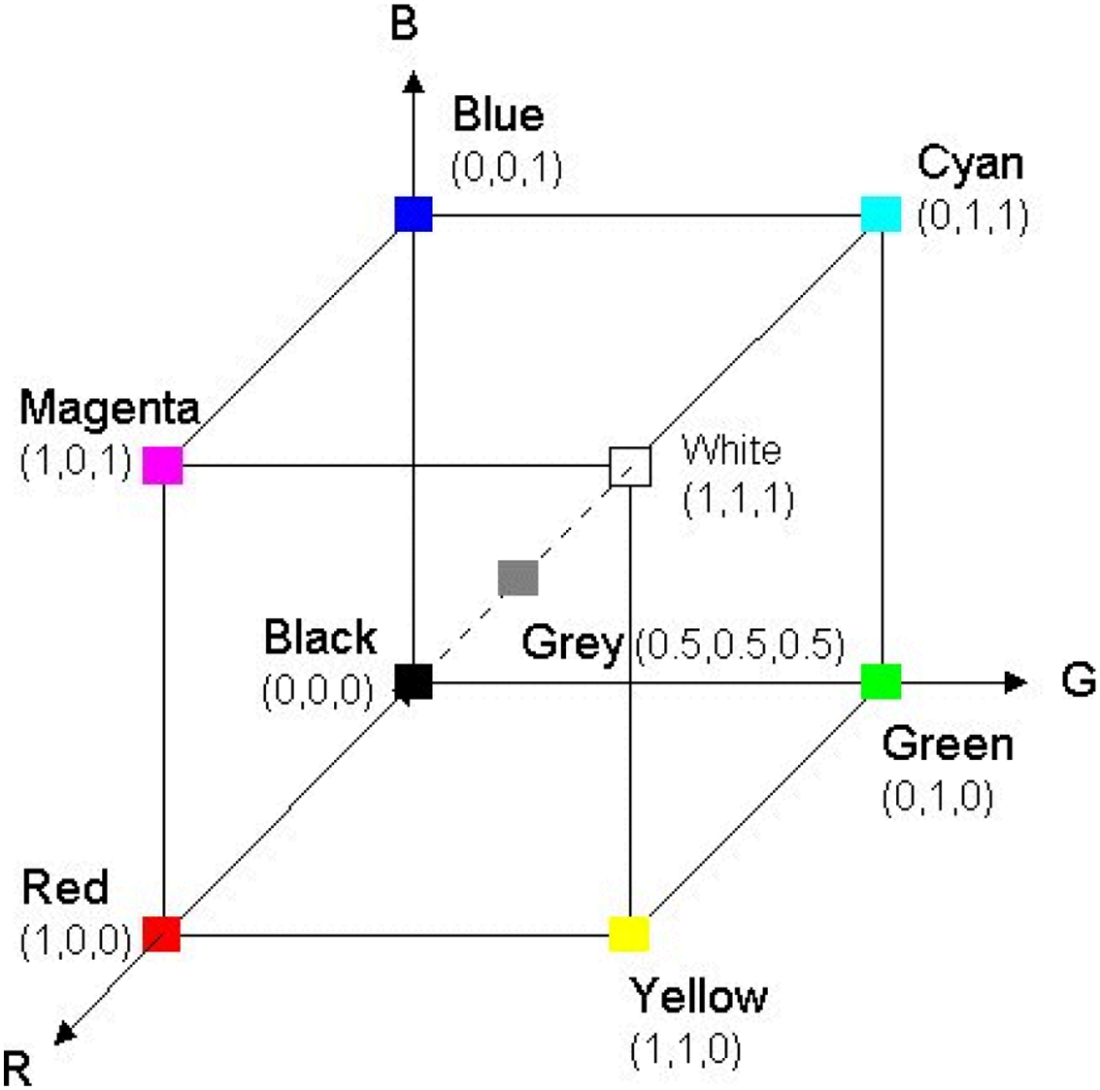
RGB representation.

RGB color space may be a broadly utilized color space for image description. It includes three color elements red, green, and blue. Its representation consists of 24-bit usage whereby 8 bits are allocated to each filter R, G, and B respectively. This means that each channel incorporates an extension of values between 0 to 255, as shown in Fig. 2, RGB representation. One remarkable shortcoming of the RGB color space is that it isn’t perceptually uniform implying that the determined separation in RGB space does not genuinely relate to the perceptual color distinction (Chavolla et al., 2018).

#### HSV color space

HSV stands for hue, saturation, and value and it was developed with the aim of determining the human perspective of colors.

It relates color (hue) ranging from 0 to 360, saturation (shade) ranging between 0–100 percent and sometimes is referred to “purity” and brightness (value) ranging between 0–100 percent. HSV is cylindrical geometry, with hue, their precise measurements, beginning at the red key at 0°, going through the green essential at 120° and the blue essential at 240°, and afterward to red at 360° (Sonka & Hlavac, 2008). The representation of HSV space is three-dimensional as a descending pointing hexacone. The line that runs down the focal point of the cone’s vertical axis constitutes the intensity of V. The point comparative with the red axis opposite to the intensity axis constitute the hue. The point’s opposite distance from the intensity axis represents saturation. The formula Eq. (1) transform from RGB to HSV (Shidik et al., 2013) is:

$$H = \; \left\{ {\matrix{ \theta \cr {{{360}^0}} \cr } } \right.\matrix{ {if\; b\; \le g} \cr {if\; b\; > g} \cr } \; \; \; \; \; \; \; \; \; \; \; \; \; \; \; \; \; \; \; \; \; \; \; \; \; \; \; \; \; \; \; \; \; \; \; \; \; \; \; \; \; \; \; \; \; \; \; \; \; \; \; \; \; \; \; \; \; \; \; \; \; \;$$

where

(1) $${\eqalign{\theta  = co{s^{ - 1}}\displaystyle{{\displaystyle{1 \over 2}\left[ {\left( {R - G} \right) + {\rm \; }\left( {R - B} \right)} \right]} \over {\sqrt {{{\left( {R - G} \right)}^2} + {\rm \; }\left( {R - B} \right)} \left( {G - B} \right)}} \cr S = 1 - {\rm \; }\displaystyle{3 \over {R + G + B}}\left( {min\left( {R,G,B} \right)} \right) \cr V = \displaystyle{1 \over 3}\left( {R + G + B} \right)}}$$

The R, G, B address red, green and blue parts individually with a value between 0–255. To acquire the estimation of H from 0° to 360°, the estimation of S and V from 0 to 1, the following equation is executed:

$$\rm {H} = {(H/255*360)\, mod\, 360}$$

$$\rm V = V/255$$

$$\rm S = S/255$$

#### LAB color space

CIE (Commission Internationale de lclairage) LAB is another very popular color space. The CIELAB color space (referred to as CIE L*a*b* or occasionally shortened as “Lab” color space) is a color space characterized by CIE (International Commission on Illumination) in 1976.

The CIE created the first color models based on the physic aspect of the light. The most notable models are, the CIE RGB, CIE XYZ, and CIELab. All of them describe how all the colors can be formed on a plane with a given illumination level. The CIE XYZ and CIE RGB are calculated by using the light wavelength from the physic representation of the color, while CIELab is indirectly obtained from the CIE XYZ (Sonka & Hlavac, 2008).

This color space is much of the time utilized as an exchange design while overseeing different hardware since its gadget autonomous. CIELAB color space communicates color with three qualities: L* representing the lightness from black (0) to white (100), a* from green (−) to red (+), and b* from blue (−) to yellow (+). CIELAB was planned with the goal that similar measure of mathematical change in these qualities compares to the general a similar measure of outwardly perceived change.

It is planned to be viable with “CIELAB” similar measure of progress in these mathematical qualities with practically a similar measure of progress seen visually (Al-saleem, Al-Hilali & Abboud, 2020). The overall numerical equations is as follows:

$$M\; \; \; \; = \; \; \matrix{ L \cr {{M_{CAT02}}} \cr L \cr } \; \; \; \; \; \; \; \matrix{ X \cr Y \cr Z \cr } ,$$

(2) $${M_{CAT02}} = \matrix{ {0.7328} \cr {0.7036} \cr {0.0030} \cr } \; \; \; \; \; \; \matrix{ X \cr Y \cr Z \cr } ,\; \; \; \; \; \; \; \; \; \; \matrix{ { - 0.1624,} \cr {0.0061,} \cr {0.9834,} \cr }$$

$$Lc = \left( {\displaystyle{{YwLwr} \over {YwrLw}}\; D + 1 - D} \right){L}^{\prime},$$

$$Mc = \left( {\displaystyle{{YwMwr} \over {YwrMw}}\; D + 1 - D} \right){M}^{\prime},$$

$$Sc = \left( {\displaystyle{{YwSwr} \over {YwrSw}}\; D + 1 - D} \right){S}^{\prime}.$$

Yw/Ywr constitute the two bright components that have similar color however are not the same as whites. Basic qualities/values demonstrate the reaction/response of the cone to white below (w) and reference radiation (wr). The change degree (D) is set to zero for non-change (self-lighting incentive) and the module for a full change (colorfastness) (Al-saleem, Al-Hilali & Abboud, 2020). Practically, the values ranging from 0.65 to 1.0, is determined by the Eq. (3).

(3) $$D = F\; \left( {1 - \; \displaystyle{1 \over {3.6}}{e^{ - \left( {{L_A} + 42} \right)/92}}} \right),$$

where surround “F” is defined above and “ ${L_A}$” is the adapting field luminance in cd/m^2^.

**Texture**

Texture in the domain of processing images provides data about the neighborhood spatial groupings of colors or the color intensities of the images. Images which have comparative texture features ought to have similar spatial groupings of colors or intensities, yet not really similar colors. Due to this, using text-based image indexing and the approaches used in retrieving is different from the techniques that uses purely colors. The texture of an image is a set of measurements that are determined in image processing for quantifying the regarded texture of an image. The texture provides details about the spatial grouping of color and intensities in an image or the chosen region of an image. Gabor filter is one of the techniques that extract image features using texture classification.

A Gabor channel is a linear channel that is characterized as a consonant/sinusoidal function multiplied by Gaussian function. The Gabor channel is a sort of bandpass channel that has some expertize in discovering characteristics like edges within an image. The channel regulates over an image in extracting characteristics from various points. This channel is the best filter that can be used prior to training and also during the training (Kamarainen, Kyrki & Kalviainen, 2002). Gabor put together his work with respect to the mechanical wave hypothesis and in Heisenberg’s vulnerability standard. He suggested that signals can be represented as a mix of these basic functions. Subsequent work covered the analysis of the specific characteristics obtained from Gabor filters, global Gabor features and basic frequency Gabor characteristics (Hamouz et al., 2003).

Gabor filter has been investigated widely in different applications such as recognizing face, classifying texture as this portray the spatial recurrence structure in images while maintaining relationships in the spatial data. This makes it possible to separate the recurrent contents from the patterns. Additionally, manageable and adaptable parts like the functions in Gabor are indicated easily and its computation is effective (Luan et al., 2018).

One of the fundamental benefits of 2-D Gabor channel is their relationship with specific area in space. Daugman has revealed that Gabor space representation reduces the joint-2-D uncertainty guideline in space and frequency. Besides, proof has as shown that the 2D open field profiles of basic cells in the mammalian visual cortex are all around portrayed by individuals from the Gabor 2-D channel family. Using the Gabor channel is very beneficial as it provide ability of the filter to provide some level of invariance, interpretation and direction. Consequently, Gabor channels are finding expanding utilization in numerous application, for example, enhancing fingerprint images, classifying and segmenting textures, recognizing images and motion tracking. Gabor channels have spatially localized administrators who are involved in the analyzing images for some time at various frequencies and in various ways to the level that many images are made out of repeating occurrence of micro- patterns that makes them suitable for representation by Gabor channels. The other advantage of Gabor filters is to eliminate the dissimilarity in lighting and contrast and minimize intrapersonal dissimilarity which makes the image appearance change due to lighting condition, shadow, *etc*.

**Figure 3:**
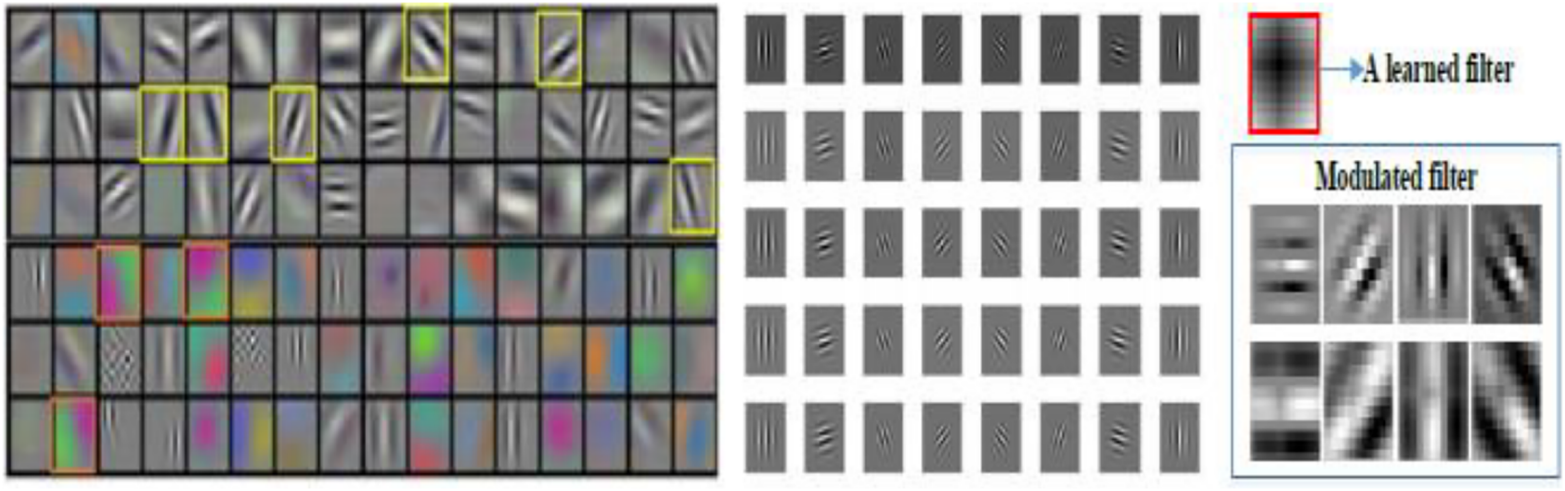
Left indicates AlexNet filters; the center indicates Gabor filters; the right shows the convolution filters regulated by Gabor filters.

Gabor filters were used where the images were converted into grayscale images and saved as a dataset. To understand how CNN learns from the image information we visualize the convolutional filters in Fig. 3 using AlexNet (Krizhevsky, Sutskever & Hinton, 2012). The convolutional filters are show from first layer. The yellow boxes in the left side are learned filters features which are similar to Gabor filters. For example, the channels with various directions records into the conventional channel configuration because of their upgraded capacity of scale and direction decay of signal, which is tragically ignored in the majority of the predominant convolutional channels in DCNNs Channels are often repetitively studied in CNN whereby some are similar to Gabor channels (as indicated by the yellow boxes). In light of this perception, we are persuaded to control the learned convolution channels utilizing Gabor channels, to accomplish a packed profound model with a decreased number of channel boundaries/parameters. From this thought, this motivates us to exploit the already absorbed convolution channels using Gabor channels to attain a compacted deep model with a lesser channel parameters. In the right column, a convolution filter is regulated by Gabor filters (Luan et al., 2018).

Our approach utilizes Gabor channel-based highlights rather than the raw gray values as the input for a convolutional neural network. Gabor eliminates the vast majority of the dissimilarity in lightning and contrast and reduces interpersonal dissimilarity. Consequently, they are robust compared to tiny shifts and tiny object deformations. The rest of the paper is coordinated as follows. In the next section, we discuss the related work. In “Research Methodology”, the components and details of the convolutional neural network and Gabor filters are presented. “Experimental Research, Analysis and Findings”, reports results that were obtained in experiments. Finally, some conclusions follow in the last section.

## Related work

Among the current spatial data extraction procedures, Gabor separating has pulled in a great deal of consideration because of its ability to give discriminative and useful highlights (Bovik, Clark & Geisler, 1990). Contrasted to other filtering techniques, Gabor filtering shows its benefits in spatial data extraction including edges and surfaces (Randen & Husøy, 1999). Gabor channels are a well-known in the extraction of the spatial localized data (Jain, Ratha & Lakshmanan, 1997).

Recurrence and direction portrayals of Gabor channels resemble those of the human visual framework, and they are explicitly fitting for surface portrayal and segregation. A particular advantage of Gabor channels is their degree of invariance to scale, revolution, and interpretation. Indeed, Yosinski et al. (2014) have shown that profound neural organizations prepared on pictures will in general learn first layer highlights looking like Gabor channels. This further verifies our instinct of utilizing pre-planned Gabor channels as weight parts in a CNN setup.

In this section, several methods that use Gabor Convolutional Networks have been used. In Caldern, Roa & Victorino (2003), character images from MNIST dataset are first subjected to extraction of Gabor features. The features extracted are then fed to basic CNN. With the MNIST dataset of 60,000 image instances, the system produced a recognition efficiency of 99.32. In Kinnikar, Husain & Meena (2016) initially Gabor features of four orientations are extracted thereby creating four images of different orientations. All four images are then separately passed to CNN for learning. Experiments with the AT & T database show efficiency of 89.50 percentages. In Xu et al. (2016), the derived Gabor features are analyzed with CNN and recognition accuracy of 93 percentages is claimed.

In Sarwar, Panda & Roy (2017), the convolution layer in CNN (with two sets of convolution layers) is replaced with Gabor kernels to increase the recognition accuracy, computational time, and energy requirements. Three variations of analyzing the convolution kernels with Gabor features were analyzed. All the variations analysed Gabor features with 12 orientations. The first variation replaced only the first convolution layer with fixed Gabor kernel values but the kernel values are never relearned. This variation obtained a recognition accuracy of 99.38 percentages with 60,000 instances of MNIST dataset. The second variation replaced the convolution kernels in first and second layer with fixed Gaussian kernels without relearning. The accuracy of the system was reduced to 94.15 percentages. The third variation replaced some of the kernels in each convolution layer with Gabor filter values and others with random initialization of values which is further relearned. The efficiency of this variation is 99 percentages.

A couple of works have related Gabor filters to DCNNs. Although, they do not explicitly coordinate Gabor channels into the convolution filters. In particular, Hu et al. (2016) basically utilizes Gabor channels to create Gabor properties and utilizes them as input to a CNN, and Hinton et al. (2012) only uses the Gabor channel in the first or second convolution layers to reduce the training complexity of CNNs.

## Research methodology

**Figure 4:**
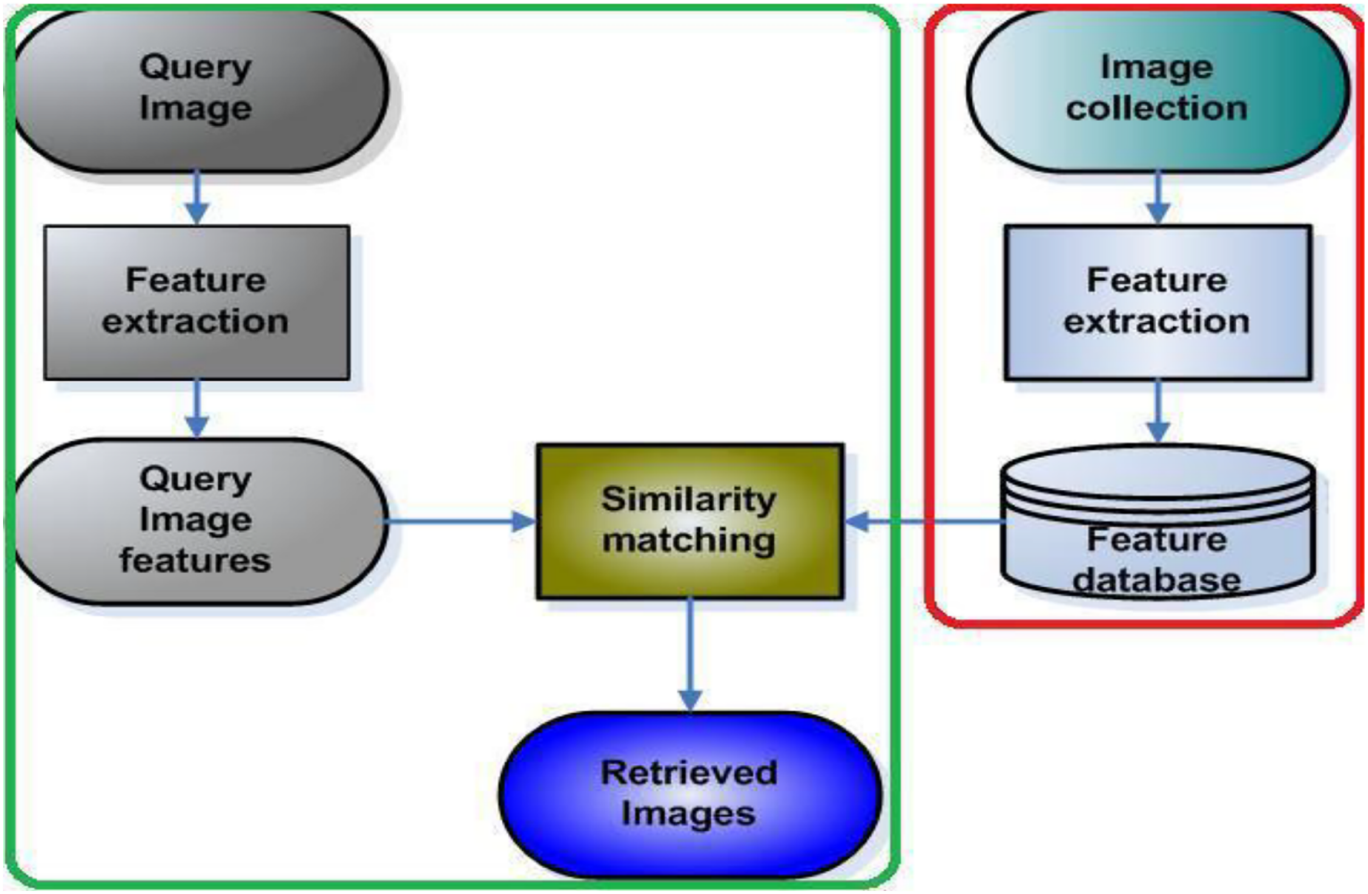
Framework of the proposed method for image retrieval.

The proposed method has two steps, first feature extraction using color spaces followed by Gabor CNN. Figure 4 demonstrates the proposed framework on image features are extracted using color space and texture and how the query is done using CNN. The image features are extracted using RGB, HSV, LAB combination of RGB+HSV, RGB+LAB, and HSV+LAB. The second step is extracting those features with the incorporated Gabor filter and CNN. The test and training images are the query image and image collection extracted using different color space and Gabor filter respectively. The similarity matching between query features and database features are done using CNN. The retrieved images are measured using precision, recall and F1score.

To begin with querying, images are extracted using color and Texture features.

**A feature extraction**

As mentioned earlier in this paper features are extracted using different color spaces as explained below.

**RGB color space**

Images in Cifar 10 dataset are RGB format. There is no need for transforming the images and therefore the features were extracted.

**HSV color space**

The transformation from RGB to HSV is shown below. R, G, and B are the values of the red, green c, and blue channels respectively. H gotten indicates the hue channel. Likewise, S indicates the saturation channel and V the value channel as in Eq. (1).

**LAB color space**

LAB is another common color space. This color space is often utilized as an interchange format when managing various gadgets.

This is done on the grounds that it is gadget autonomous. Here, L represents lightness, ‘a’ represents color component green-red, and ‘b’ represents blue-yellow. Eq. (3) was used to transform.

**GaborNet**

To improve the retrieval efficiency Gabor filter was introduced. A benefit of Gabor filters is their level of invariance to scale, rotation, and translation. The impulse response is a product of a sinusoidal function and a Gaussian function. The Gabor filter coefficients are calculated as

$$g\left( {x.y;\lambda ,\theta ,\psi ,\sigma ,\gamma } \right)$$

(4) $$= exp\left( { - \displaystyle{{{x^2} + {\gamma ^2}{y^2}} \over {2{\sigma ^2}}}} \right)exp\left( {i\left( {2\pi \displaystyle{{{x^1}} \over \lambda } + \psi } \right)} \right)$$

The list of equations symbols and meaning for Eq. (4) is elaborated in Table 1.

**Table 1:** List of equations symbols and meaning.

Symbols	Meanings
γ (Gamma)	Aspect ratio
*λ* (Lambda)	Wavelength
σ (Sigma)	Scale
θ (Theta)	Orientation of Gabor kernels

The range of x and y determines the size of the Gabor filter. The size of the filter has a direct effect on building an efficient recognition system. Even though deep learning is trying to build a recognition system that is applied numerous applications, the selection of a generalized size filter may reduce the efficiency of the recognition system. Too large and too small size may have an inverse effect in recognition systems. Large filters may sharpen the noise in the recognition system and small-sized filters for useful information. γ is the aspect ratio, and *λ* determines wavelength. These parameters together determine the frequency change (rate of change of adjacent values) in kernel data distribution in both directions of a two-dimensional kernel. A smaller wavelength means a denser sinus wave. A larger wavelength means larger waves.

σ determines the scale (effective width). Data values in the kernel are randomly distributed in such a way that variance of data should be of a particular scale σ. Larger values of σ capture a broader range of frequencies resulting in tighter bandpass and a poorer spatial localization. By selecting a different range of σ values the Gabor filter tends to work in different scales.

θ determines the orientation of Gabor kernels. The orientation aspect of Gabor kernels makes object recognition using the Gabor filter invariant to rotation. As Gabor filters are a combination of sinusoidal wave and Gaussian function σ and θ determines Gaussian component and γ and *λ* determines sinusoidal component (Jyothi, Rahiman & Anilkumar, 2018).

The CNN architecture is split into four stages. The first two stages consist of Convolution Layer, RELU Layer, and Max pooling Layer. The third stage consists of Convolution Layer and RELU Layer. The last fourth stage consists of a fully connected layer. The first stage of the Convolution layer is initialized with Gabor Kernels and the other layers take the default architecture of CNN.

The first convolution layer is designed based on 16 Gabor filters with uniformly distributed θ (orientation) values. A Gabor kernel of size 3 × 3 is used as convolution filter, Scale (σ) as 4.1, γ as 5.3 and *λ* as 0.3. Selection of all the values is based on experimental analysis. The first Convolutional layer converts a single input image into 16 output images which are passed to the RELU layer.

The second Convolution Layer and third stage work on kernels initialized with random Normal distribution values with a standard deviation of 0.0001. Biases are initialized with Normal distribution of mean value 1 and standard deviation 0.00001. The learning rate factor of the bias is set to 2. A total of 32 and 64 filters are used in the first and second Convolution layer respectively.

Rectified Linear Unit (ReLU) layer performs a threshold operation to each element, where any input value less than zero is set to zero. ReLU Layer is utilized in the initial three stages.

Max pooling layer performs max-pooling with a kernel size of 2 × 2 that works with a stride of ‘2’ is used in the first and second Layers.

A fully connected layer is initialized with Normal distribution with a mean 0 (Zero) and standard deviation of 0.0001 for the weight values. Bias values are initialized with normal distribution with a mean of 1 and deviation of 0.0001. The learning rate factor of the bias is set to 2. Softmax is used as an activation function in the output layer of the fully connected layer.

## Experimental research, analysis and findings

The performance of the proposed model is evaluated using Cifar 10 datasets in this section.

**ALGORITHM**

- Create a database of normalised images of 32 $\times\ 32$ in size.
- Read the Query input image which are in RGB format.
- Transform RGB images to different color space format of HSV and LAB and combination of color space.
- Use image features from different color space as an input to Gabor CNN which is the first layer.
  - To get input image :-
  - For each frequency list γ and *λ*
  - For each scale list σ – 5 scales {0, 1, 2, 3, 4}
  - For each orientation list θ – 8 Orientation {0, 1, 2, 3, 4, 5, 6, 7}
  - Construct Gabor filter (sinusoidal wave (γ and *λ*) and Gaussian function (σ and θ))
  - Convolve Gabor filter with original image to get Gabor input image
- Compute the features using the same techniques for Image database.
- Query image feature from the features of the image database using CNN model.
  - To query image :-
  - While not converge Do
  - For each image Do
  - Compute the convolution features from Gabor input image yℓij = σ(xℓij).
  - Apply activation function to map them in non-linear space (ReLU−f(x) = max (0, x))
  - Continue forward pass and backpropagation to learn model parameters (∂xℓ(i−a)(j−b)∂yℓ−1ij = ωab∂x(i−a)(j−b)ℓ∂yijℓ−1 = ωab)
  - End for
  - End while
- Retrieves similar images from the image database.
- Compute and display the percentage similarity of the retrieved images.
- Calculate Precision, Recall, F1score, Receiver Operator Characteristic (ROC) and Area Under Curve (AUC).

**Recall and precision calculation**

Testing the effectiveness of the retrieval is about testing how effective the search engine can retrieve similar images and how the model avoids retrieval of irrelevant images.

Recall is a measure of the ability of a model to produce all relevant images (Patil, 2012). It is the number of correct images divided by the number of relevant images that should have been returned.

Precision is a measure of the ability of a model to produce only relevant images. It is the number of correct images divided by the number of total images returned.

F1score is a measure of test accuracy. It is the average of precision and recall.

ROC uses different probability thresholds to summarize the trade-off between the true positive and false positive rate for a predictive model.

Area Under Curve summarizes the approximation of the area under the precision and recall curve.

**Gabor parameters**

The size of the Gabor filter is designed depending on analysis of the experiments the efficiency of the system is tested with Gabor filters of different sizes. The system is tested with Gabor filters of sizes 3 × 3, 5 × 5, and 7 × 7. The highest efficiency has been produced when the kernel size is 3 × 3. When the size of the filter is 3 × 3 the efficiency of the system is 99.68 and when the kernel size is 5 × 5 the efficiency of the system is 98.69 and when the size of the kernel is 7 × 7 the efficiency of the system is 98.46. Therefore kernel size of 3 × 3 is selected. The selection of σ, γ and *λ* is also carried out based on experimental analysis. It can be found that when σ is 4.1, γ is 5.3 and *λ* is 0.3 the system produces the highest recognition efficiency. Combinations of different values of σ and γ ranging between 1 and 10 are tested for the system and it is found that σ values between 4 and 5 and γ values between 5 to 6 produces highest recognition rate. Again testing of the recognition system is repeated with different combinations of σ between 4.1 to 4.9 and γ between 5.1 to 5.9 and it is found highest efficiency is reported when σ is 4.1 and γ is 5.3. Fixing σ as 4.1 and γ as 5.3 different *λ* values ranging from 0 to 0.5 is tested and it is found that fixing *λ* values to 0.3 will increase the efficiency of the system. For the selection of parameters of Gabor filters testing is carried out in a subset of the CIFAR10 dataset.

**Training CIFAR10 dataset**

The research used the CIFAR 10 dataset which is one of the most generally utilized datasets for AI research.

The CIFAR-10 dataset contains 60,000 natural images of 10 different classes represented as airplanes, cars, birds, cats, deer, dogs, frogs, horses, ships, and trucks. The images are of 32 × 32 size. The dataset is split into test and training clusters containing 1,000 and 5,000 images respectively. The test images are randomly selected from each class but same number of images from each class. The remaining images are training which are in random order, some training batches may contain more images from one class.

The classes are totally fundamentally unrelated. There is no overlap between automobiles and trucks. “Automobile” includes sedans, SUVs, things of that sort. “Truck” includes only big trucks. Neither includes pickup trucks (CIFAR-10 and CIFAR-100 datasets. (n.d.) https://www.cs.toronto.edu/~kriz/cifar.html).

The experiments were carried out and an efficiency of 99.68 percentage has been reported.

**Architecture**

A simple CNN network consisting of four stages is created with the first three stages consists of convolution layer followed by ReLU layer. Convolution ReLU layers in the first and second stage are followed by the max-pooling layer. Convolution kernels of the first convolution layer are made of 16 Gabor kernels initialized with a random normal distribution and values (0, 12, 24, 36, 48, 60, 72, 84, 96, 108, 120, 132, 144, 156, 168, 180). A Gabor kernel of size 3 × 3 is used as convolution filter. A single column and row of padding with zero values is done in the top, bottom, left and right of the input image before performing convolution. The convolution layer of the first group convert a single input image into 16 output images which is then passed to the ReLU Layer.

Convolution Layer in second and third group works on kernels initialized with random Gaussian distribution values with standard deviation 0.0001. Thirty two filters are used for convolution in the first group and 64 filters are used for convolution in the in the second group. Biases are initialized with Gaussian distribution of mean value 1 and standard deviation 0.00001.

The learning rate is set to 0.01. Rectified Linear Unit (ReLU) layer returns 0 wherever it receives a negative input value and returns X value for a positive received value of X. It is used in the first three groups.

Maxpooling layer calculates the maximum value from the region of the feature map covered by the kernel. The kernel size is set to 2 × 2 to works with a stride of ‘2’ in the first and second group.

The last layer is fully connected layer with output size 10. In this layer softmax activation is used, the weight values initialized with Gaussian distribution with mean Zero and standard deviation 0.0001. The learning rate set to 0.01. The bias values are initialized with Gaussian distribution with a mean of 1 and deviation of 0.0001.

The architecture is trained using cifar 10 dataset with 60,000 images of each dataset where, out of which 10,000 used for training and others are used for validation and testing.

Experiments are done for comparing our approach with the state-of-the-art networks.

Table 2 shows the summary of a simple CNN model. The experiment started with RGB, HSV, CIELab, RGB + HSV, RGB + CIELab, HSV + CIELab respectively and done at 50 Epochs. The Accuracy, Mean Average Precision (MAP), and Mean Average Recall (MAR) are as follows:-

**Table 2:** Summary of a simple CNN model.

	RGB	HSV	LAB	RGBHVS	RGBLAB	HSVLAB
Accuracy %	73	69	69	69	67	63
MAP %	73	70	69	69	69	65
MAR %	73	69	69	69	67	63

Table 3 shows the summary of color space combined with Gabor filters.

**Table 3:** Summary of color space combined with Gabor filters.

	RGB	HSV	LAB	RGBHVS	RGBLAB	HSVLAB
Accuracy %	99.78	99.69	99.68	99.69	99.69	98.89
MAP %	99.78	99.69	99.67	99.73	99.71	98.89
MAR %	99.78	99.67	99.67	99.69	99.69	98.88

Receiver Operator Characteristic (ROC) plots were used for each classifier and their classes to investigate the trade-off between true positive and false positive rate. Accuracy rate will also be carried out to measure retrieval rate.

**Figure 5:**
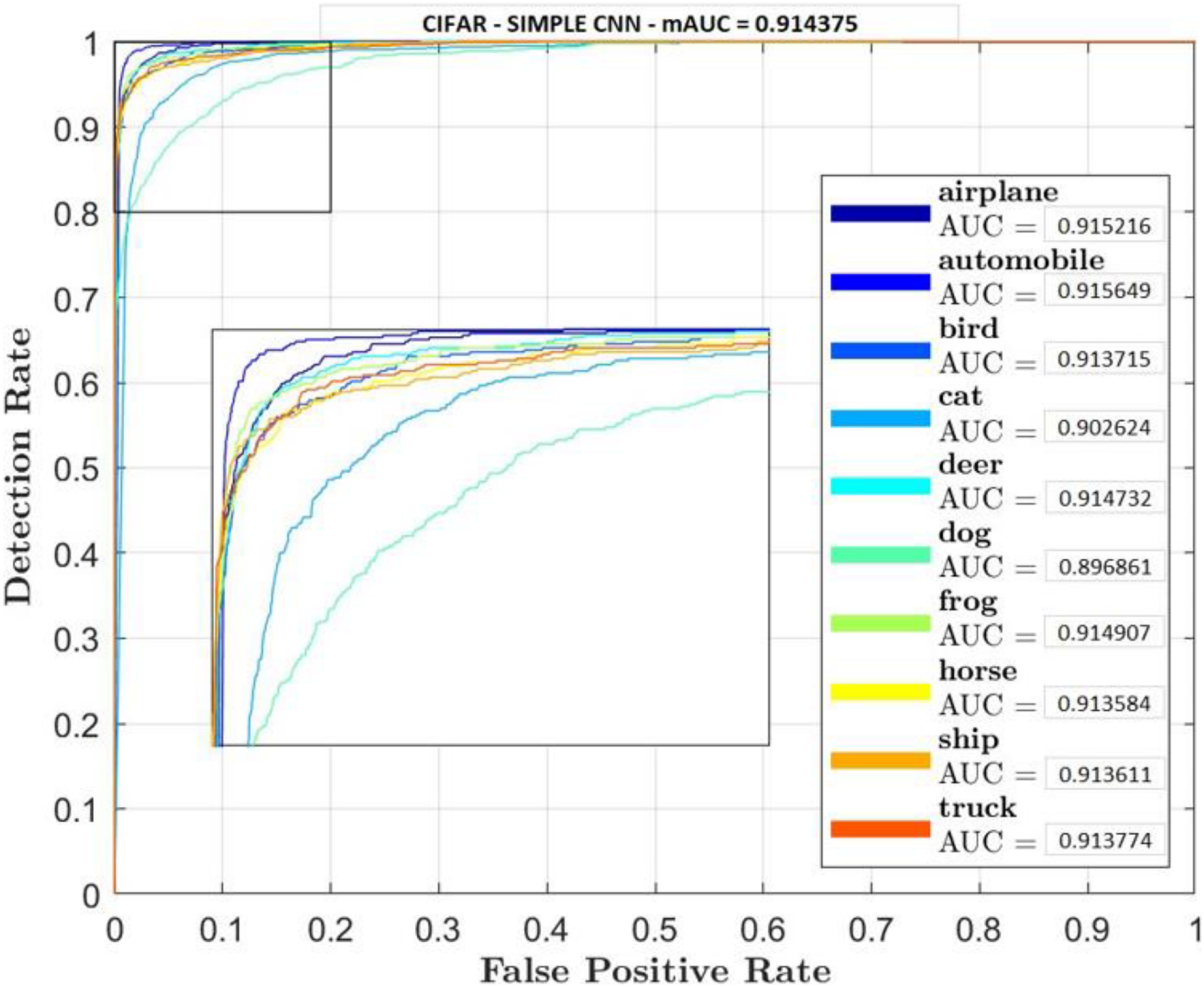
Simple CNN.

**Figure 6:**
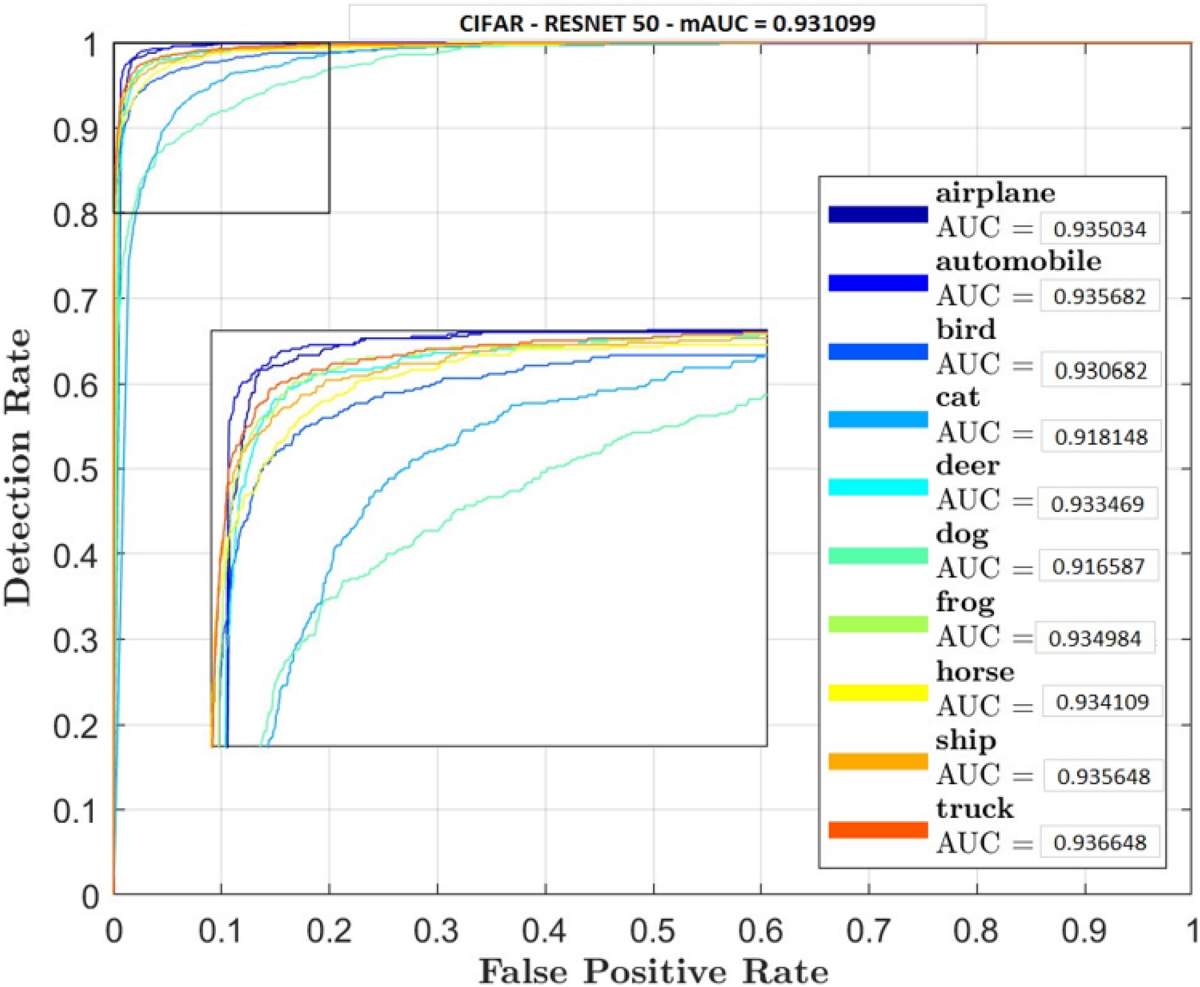
ResNet 50.

**Figure 7:**
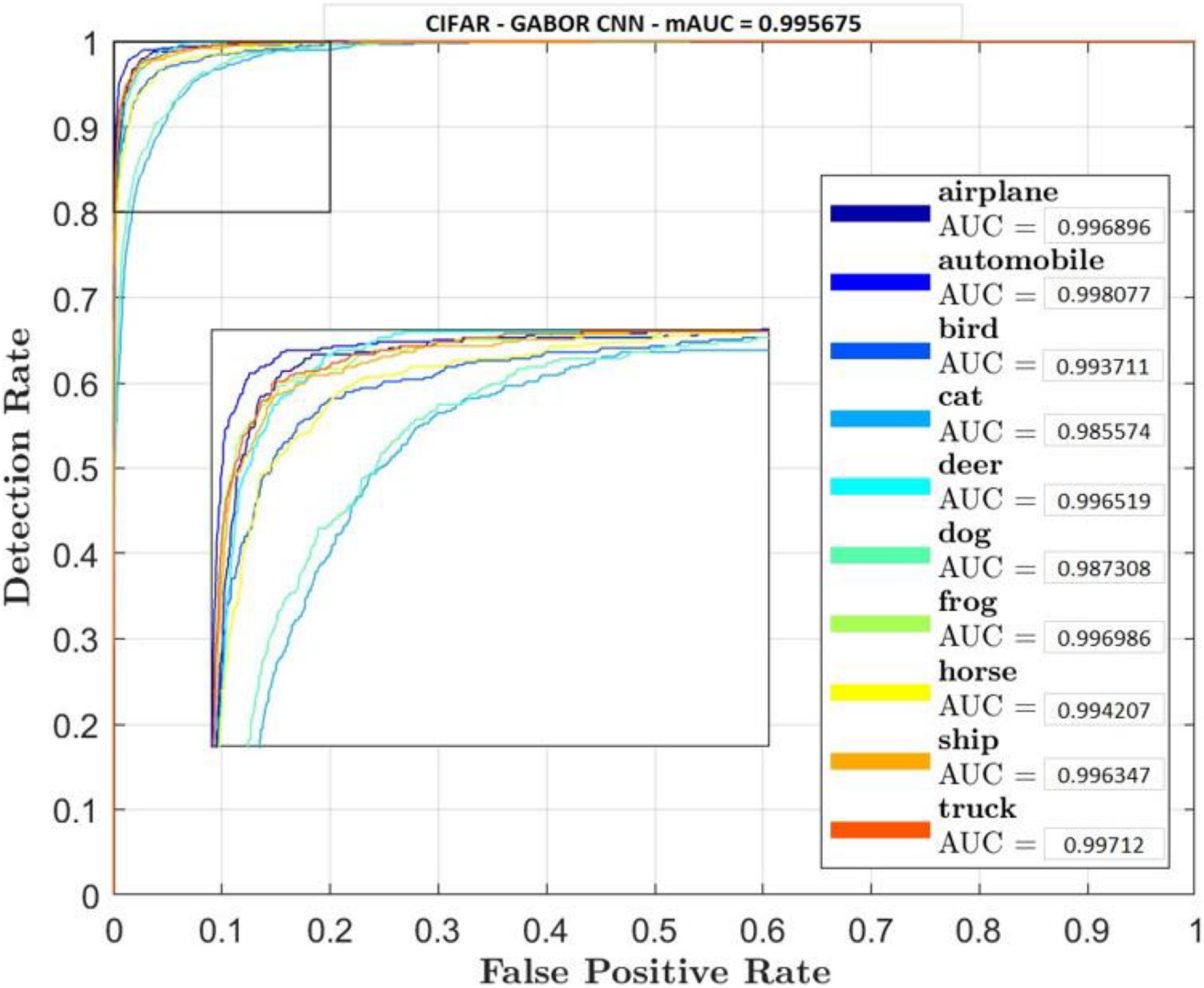
Gabor CNN.

Figures 5–7 shows ROC curve and AUC curve for simple CNN, ResNet 50 and Gabor CNN respectively.

Table 4 shows that GaborNet reliably improves the introduction paying little heed to the quantity of boundaries/parameters or kernels as contrasted and the simple CNN and ResNet 50. The proposed network has a predominant presentation in Feature Extraction and arrangement. In this context, the GaborNet achieves higher order exactness/precision than that of CNN and ResNet when color spaces were used.

**Table 4:** Comparison of Simple CNN and GaborCNN color space.

Color space	Simple CNN	GCN
RGB	78.89	96.99
HSV	78.57	96.86
RGBHSV	–	96.85
YUV	78.89	–
LAB	80.43	96.88
RGBLAB	–	96.84
HSVLAB	–	96.83
YIQ	78.79	–
XYZ	78.72	–
YPbPr	78.81	–
YCbCr	78.81	–
HED	78.98	–
LCH	78.82	–

Findings

Two comparative experiments are conducted, simple CNN and GaborNet and experimental results are listed in Tables 2 and 3 respectively. Both the models were tested using same hyper parameters. Simple CNN model shows, RGB, LAB and RGBHSV has high performance metric although their Accuracy is low compared with proposed GaborNet model. In GaborNet the accuracy is high and also the variance between the three metrics Accuracy, Precision and Recall is unnoticeable. At closer look at confusion matric its noted different classes are effectively retrieved using different color space combined with Gabor filter. Comparing the results with simple CNN and state-of-art ResNet 50 model which produced 91.4% and 93.1% accuracy respectively, GaborNet prove more improved. The results shows when HSV and any combination of HSV color space is used there is increase in accuracy retrieval for bird, cat, deer and dog. Although the accuracy of the model still remains the same. The results also shows when LAB and any combination of LAB color space is used there is increase in accuracy retrieval for frog, horse, ship and truck.

## Conclusion

The study found that preprocessing images by changing them into various color spaces produced various outcomes. Though, the precision/accuracy by itself didn’t fluctuate excessively, after looking into it further with the guide of confusion matrices and ROC curve we found that there was not a 100% correlation between outcomes. We found out that different color space retrieve effectively different images.

The research concluded that the proposed GaborNet (Gabor Convolutional Networks) improve retrieval of images. The broad investigations indicates that GaborNet essentially improved baselines, bringing about cutting edge execution more than a few benchmarks. The research concluded that the discrepancy is due to the position of the object with respect to the camera where some aspects of the object hides and the computer see different numbers. The research also concluded, Gabor CNN perform more better on the categories with large scale variances like bird, cat, beer, and ship Besides this is because of loss of data brought about by the projection of the 3D world on a 2D image. Taking all the previously mentioned factors into thought, we can infer that the proposed GaboNet strategy has a prevalent benefit in feature extraction with color spaces alone.

## Supplemental Information

10.7717/peerj-cs.890/supp-1 Supplemental Information 1 Pure Gabor.

10.7717/peerj-cs.890/supp-2 Supplemental Information 2 cifar Gabor.

## Additional Information and Declarations

### Data Availability

The following information was supplied regarding data availability: Dataset link https://figshare.com/s/0c1dfc3be66eb622cf85 - cifar 10. The code is available in the Supplemental Files.
